# HIV-1 gp41 Core with Exposed Membrane-Proximal External Region Inducing Broad HIV-1 Neutralizing Antibodies

**DOI:** 10.1371/journal.pone.0018233

**Published:** 2011-03-31

**Authors:** Ji Wang, Pei Tong, Lu Lu, Leilei Zhou, Liling Xu, Shibo Jiang, Ying-hua Chen

**Affiliations:** 1 School of Life Sciences, Tsinghua University, Beijing, China; 2 New York Blood Center, Lindsley F. Kimball Research Institute, New York, New York, United States of America; 3 Key Laboratory of Medical Molecular Virology of Ministry of Education/Ministry of Health and Institutes of Biomedical Sciences, Shanghai Medical College, Fudan University, Shanghai, China; Beckman Research Institute of the City of Hope, United States of America

## Abstract

The membrane-proximal external region (MPER) of the HIV-1 gp41 consists of epitopes for the broadly cross-neutralizing monoclonal antibodies 2F5 and 4E10. However, antigens containing the linear sequence of these epitopes are unable to elicit potent and broad neutralizing antibody responses in vaccinated hosts, possibly because of inappropriate conformation of these epitopes. Here we designed a recombinant antigen, designated NCM, which comprises the N- and C-terminal heptad repeats that can form a six-helix bundle (6HB) core and the MPER domain of gp41. Two mutations (T569A and I675V) previously reported to expose the neutralization epitopes were introduced into NCM to generate mutants named NCM(TA), NCM(IV), and NCM(TAIV). Our results showed that NCM and its mutants could react with antibodies specific for 6HB and MPER of gp41, suggesting that these antigens are in the form of a trimer of heterodimer (i.e., 6HB) with three exposed MPER tails. Antigen with double mutations, NCM(TAIV), elicited much stronger antibody response in rabbits than immunogens with single mutation, NCM(TA) and NCM(IV), or no mutation, NCM. The purified MPER-specific antibodies induced by NCM(TAIV) exhibited broad neutralizing activity, while the purified 6HB-specific antibodies showed no detectable neutralizing activity. Our recombinant antigen design supported by an investigation of its underlying molecular mechanisms provides a strong scientific platform for the discovery of a gp41 MPER-based AIDS vaccine.

## Introduction

As the acquired immunodeficiency syndrome (AIDS) pandemic continues to expand globally, the search for a preventive vaccine is an absolute priority in combating its causative agent, human immunodeficiency virus type 1 (HIV-1). Stimulating the humoral arms of the host immune response and designing immunogens capable of eliciting broadly neutralizing antibodies (BNAbs) is a paramount goal for AIDS vaccine development [1]. However, the ability of HIV-1 to evade host immune defenses, along with substantial genetic variation, has posed major stumbling blocks to thwart this effort [2].

**Figure 1:**
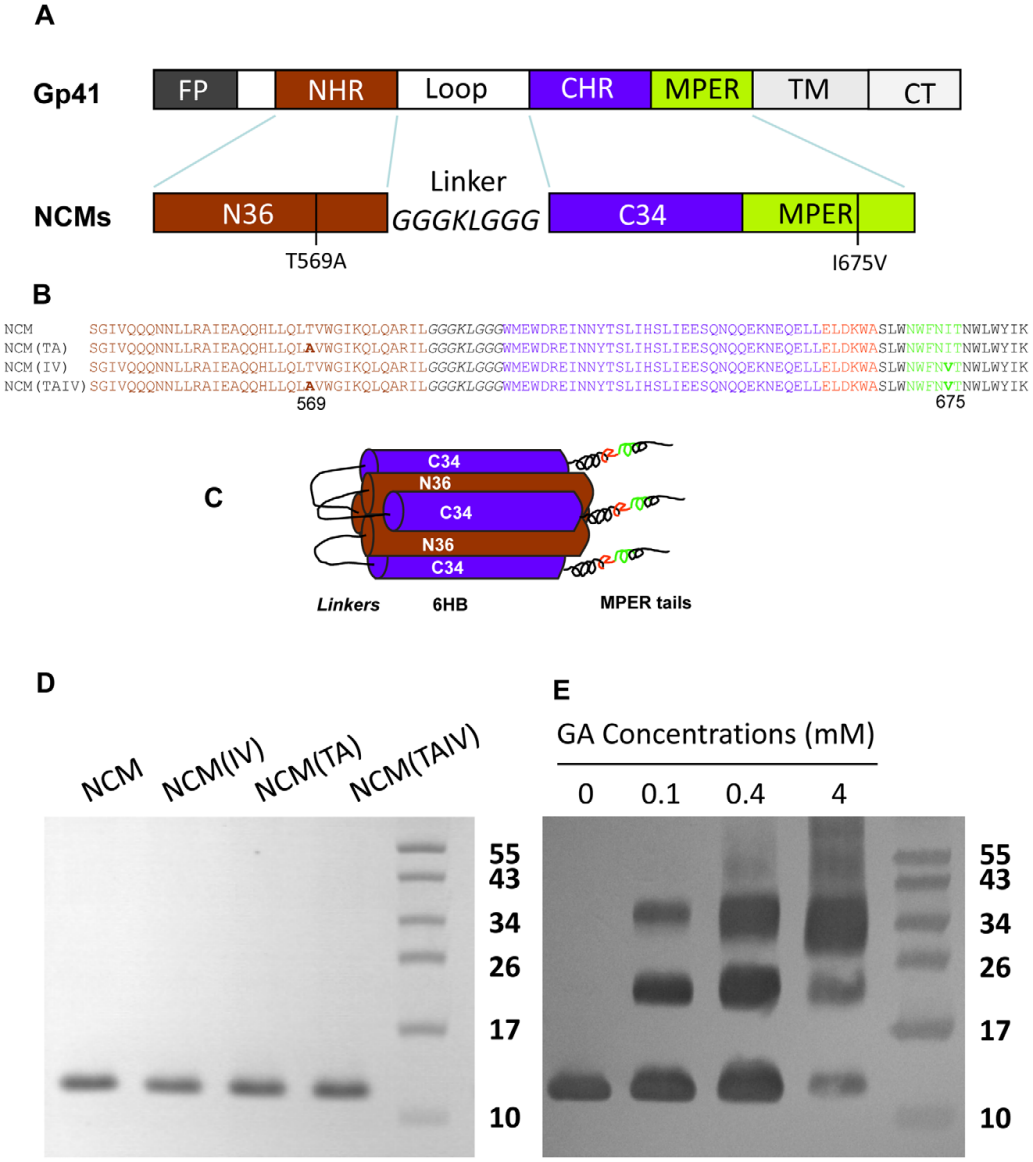
Immunogen design and protein purification. (A) Schematic representation of gp41 and NCMs. The gp41 molecule consists of fusion peptide (FP), N-terminal heptad repeat (NHR), loop (immunodominant) region, C-terminal heptad repeat (CHR), membrane-proximal external region (MPER), and cytoplasm tail (CT). (B) The amino acid sequences of NCMs. The sequence of N36 and C34 are in brown and blue, respectively. The epitopes for 2F5 (ELDKWA) and 4E10 (NWFDIT) are highlighted in red and green, respectively. The mutations of T569A and I675V are in boldface. (C) NCMs may form 6HB with exposed MPER tails. The epitopes for 2F5 and 4E10 are in red and green, respectively. (D) SDS-PAGE analysis of purified NCMs. The estimated molecular weight of NCMs is 11.8 kD. (E) Chemical cross-linking assay of NCM. NCM(IV), NCM(TA) and NCM(TAIV) showed similar results (data not shown).

Clinical studies indicate that the antisera of some HIV-1-infected patients exhibit broad and potent neutralizing activity [3], [4]. Nevertheless, only a handful of BNAbs have been identified to date. Three of those, monoclonal antibodies (mAbs) 2F5, 4E10 and Z13, target the adjacent linear epitopes located in the membrane-proximal external region (MPER) of gp41 [5], [6], which plays a crucial role in membrane fusion and viral entry. The gp41 ectodomain contains the N- and C-terminal heptad repeats (NHR and CHR), which can interact with each other to form a six-helix bundle (6HB, also known as a trimer of heterodimers), a fusion core conformation of gp41 (Fig. 1A). The mAb 2F5 recognizes a core epitope of six amino acids ELDKWA (aa 662–667) in the MPER [5]. The core binding motif, DKW, is in an extended β-turn conformation in complex with 2F5 antibody as shown by crystallographic study [7]. The 4E10 epitope comprises the linear sequence NWFNIT (aa 671–676) with several crucial Trp residues adjacent to the core motif [6], [8] and adopts a helical conformation [9]. The residue F673 swings 180° from membrane interior to the antibody binding pocket [10]. Before the recent identification of BNAbs PG9, PG16, VRC01 and VRC02 [11], [12], 2F5 had been the most potent neutralizing antibody, whereas 4E10 was able to neutralize the broadest range of different viral isolates [13], [14]. The relatively conserved and linear property of these neutralizing epitopes makes MPER one of the most attractive targets for development of an HIV-1 vaccine.

Unfortunately, in contrast to the loop region of gp41, the MPER is weakly immunogenic [15]. A number of immunization studies demonstrated that the MPER-containing recombinant proteins, proteins expressed on Virus-Like Particles (VLPs) and chimeric proteins all failed to elicit MPER-specific neutralizing antibodies [16], [17], [18]. Thus, designing immunogens that produce high titers of MPER-specific antibodies is the prerequisite for an MPER-based vaccine. Blish et al. have shown that introduction of two mutations (T569A and I675V) into gp41 results in significantly enhanced exposure of the conserved neutralization epitopes in MPER and renders the HIV-1 mutants 360- and 180-fold more susceptible to 2F5 and 4E10, respectively [19].

Therefore, we hypothesized that introducing these two mutations into an MPER-based HIV-1 vaccine candidate might increase its immunogenicity to elicit stronger MPER-specific antibodies with broad neutralizing activity. In the present study, we constructed and expressed a recombinant immunogen consisting of the N- and C-terminal heptad repeats that can form a six-helix bundle (6HB) and the MPER region of gp41. Two mutations (T569A and I675V) previously reported to expose the neutralization epitopes were introduced into NCM to generate mutants named NCM(TA), NCM(IV) and NCM(TAIV). As expected, relatively high titers of the MPER-specific antibodies were induced by NCM(TAIV). These antibodies exhibited broad and potent neutralizing activity.

## Materials and Methods

### Peptides, cells and viruses

All peptides, including MPER (aa 659–683), MPER(IV) (MPER peptide with I675V mutation), M1 (aa 645–663), M2 (aa 638–672), M3 (aa 662–676) and M4 (aa 671–683), were synthesized by SBS Genetech, Beijing, China, with purity >90%.

3T3 cells stably transduced with MLV MX-CD4 and MX-CXCR4 vectors (3T3.T4.CXCR4) were cultured in Dulbecco's modified Eagle medium (DMEM) with 10% fetal bovine serum (FBS) (Invitrogen, Carlsbad, CA). CHO cells stably transfected with either the HIV-1 HXB2 Env expressing vector pEE14 (CHO-WT) or control pEE14 vector (CHO-EE) were cultured in Glutamine-deficient minimal essential medium (GMEM) containing 400 µM Methionine sulfoximine (Sigma, St. Louis, MO). MT-2 and TZM-bl cells; plasmids, including pNL4-3.LucR^−^E^−^, Env- expressing plasmid pHXB2 and pJRFL; viruses, including HIV-1 strains IIIB, Bal, and primary HIV-1 isolates, were obtained from the NIH AIDS Research and Reference Reagent Program. GFP-transduced human osteosarcoma cells (Ghost) target cells which express CD4 and both coreceptors CCR5 and CXCR4 were kindly provided by Dr. Linqi Zhang at Tsinghua University.

### Protein expression and purification

NCM is composed of the NHR (aa 546–581) followed by an 8 amino acids linker (GGGKLGGG), CHR (aa 628–658) and MPER (aa 659–683). The cDNA was amplified from pcDNA3.1-gp160 (HXB2) plasmid and cloned into pGEX-6p-1 vector (GE Healthcare, Sweden). Three mutations were constructed by a standard point mutation method by PCR. The plasmids were transformed into *E. coli* strain *Rosetta*. The proteins were induced at 16°C with 1 mM IPTG for 12 hours. Purification of the proteins followed a standard procedure with glutathione Sepharose 4B Fast flow beads (GE Healthcare, Sweden). The GST-fusion proteins on beads were cut by PreScission protease (GE Healthcare, Sweden) at 4°C overnight in PPase buffer (150 mM NaCl, 100 mM Tris, 1 mM EDTA, 1 mM DDT). The beads were washed with 1 mM glutathione in PBS to remove GST (NCM bound tightly to beads). The NCMs were collected by washing beads with 6 M guanidine. All proteins were purified to homogeneity by Vydac C4 Reversed-Phase Columns (Grace, MA), lyophilized and stored at −20°C. NCMs were dissolved by PBS buffer with 0.1% triton X-100 for hydrophobic character of MPER. Expression and purification of six-helix bundle (6HB) protein composed of NHR and CHR of gp41, N36(L8)C34, was described previously [20]. Protein concentrations were quantified with bicinchoninic acid (BCA) assay.

### Chemical cross-linking and Western blot analysis

Cross-linking study was performed as described by Mische et al [21]. Briefly, NCMs were treated with 0, 0.1, 0.4 and 4 mM glutaraldehyde (GA), respectively, for 1.5 h at room temperature and were separated by SDS-PAGE. The protein bands in the gels were transferred into a PVDF membrane (Millipore) and analyzed with Western blot using the mAb NC-1 [22] as the primary antibody.

### Enzyme-linked immunosorbent assay (ELISA)

The NCMs were coated onto the microtiter plates (0.25 µg/well, pH 9.6, 0.1 M NaHCO_3_) at 4°C overnight and blocked by 0.3% gelatin (in PBS) for 1 h. Then the primary antibodies 2F5, 4E10 (Polymun, Austria) or NC-1 were added and incubated for 1 h. Thereafter, the peroxidase-conjugated anti-human (Sigma, MO) or anti-mouse immunoglobulin antibodies (Dako, Denmark) were added, and 1 h incubation was performed. The freshly prepared substrate solution (10 mg OPD in 10 ml, pH 5.0, 0.1 M citrate buffer, and 18 µl H_2_O_2_) was then added to each well. The reaction was stopped by 2 M H_2_SO_4_ 10 minutes later, and the absorbance at 490 nm (A490) was read by a Bio-Rad Model 680 (Hercules, CA).

### Surface plasmon resonance (SPR) assay

A BIAcore X instrument was used in SPR binding assays. The SPR experiment to investigate the binding of 2F5 to NCMs on membrane was performed as previously described [23]. Briefly, liposomes of a mixture of POPC/Chol/SM (1∶1∶1) were captured onto sensor chip L1 surface. After washing away the loosely bound liposomes by a NaOH pulse, 45 µl 0.6 µM NCM or mutants were injected. Then the capture levels were measured after a 400 s delay. 0.01% Triton X-100 in buffer, which was crucial for NCM solubility, was confirmed not to cause large baseline changes in this study by buffer-only injections over membrane surface (changed 51±16 RU, 0.5% of capture level of liposomes to chip surface). Injections of 2F5 (50–200 nM, 60 s) or 4E10 (100–300 nM, 96 s) were taken subsequently, followed by uniform dissociation time (600 s). The data were analyzed by BIAevaluation 3.1 in 1∶1 binding with drifting baseline model, which had lower χ^2^ values than those of a two-step binding model.

### Rabbit immunization and serological test

Twelve female New Zealand rabbits enrolled for the immunization were randomly allotted to four different groups, three for each group (Table 1). The study of animals was carried out in strict accordance with the recommendations in the Guide for the Care and Use of Laboratory Animals of the National Institutes of Health and Tsinghua University. The protocol was approved by the Committee on the Ethics of Animal Experiments of Tsinghua University (Permit Number: 2010-ChenYH-A2). All efforts were made to minimize suffering. The rabbits were immunized 3 times with 50 µg immunogen at day 0, 14 and 28 by subcutaneous injections. Complete Freund's adjuvant was used for the priming and incomplete Freund's adjuvant for the next two boosts. Pre-immunization blood samples were collected one day before the primary immunization. The terminal bleeding was done on day 35. Sera were stored at −20°C.

**Table 1:** The immunogenicity of NCMs after the third immunization.

Group	Immunogen	Rabbit	Titers of antibodies specific for		
		No	Immunogen	MPER peptide	MPER (IV) peptide
I	NCM	1	1∶4×10^5^	<1∶400	<1∶400
		2	1∶1×10^5^	1∶1600	1∶800
		3	1∶1×10^5^	<1∶400	<1∶400
Geometric mean titer			1∶1.6×10^5^	1∶635	1∶504
II	NCM(IV)	4	1∶1×10^5^	1∶800	1∶800
		5	1∶4×10^5^	1∶3200	1∶3200
		6	1∶4×10^5^	1∶400	1∶400
Geometric mean titer			1∶2.5×10^5^	1∶1008	1∶1008
III	NCM(TA)	7	1∶1×10^5^	1∶1600	1∶800
		8	1∶1×10^5^	<1∶400	<1∶400
		9	1∶4×10^5^	1∶3200	1∶3200
Geometric mean titer			1∶1.6×10^5^	1∶1270	1∶1008
IV	NCM(TAIV)	10	1∶4×10^5^	1∶12800	1∶12800
		11	1∶4×10^5^	1∶25600	1∶25600
		12	1∶4×10^5^	1∶25600	1∶12800
Geometric mean titer			1∶4×10^5^	1∶20319	1∶16127

**Note:** “<1∶400” was treated as 1∶400 when the Geometric mean titer was calculated.

The immunogenicity of NCMs and MPER in the individual immunogens was characterized by indirect ELISA. The synthetic peptide MPER or NCMs were coated onto the microtiter plates (0.25 µg/well). Sera were serially diluted and added into wells. The peroxidase-conjugated anti-rabbit immunoglobulin antibodies (Dako, Denmark) served as secondary antibodies. The OPD substrate was used and quenched by 2 M H_2_SO_4_ 10 minutes later. Titers of the antigen-specific antibodies were expressed as the highest dilution of serum that reacted with the coating antigens (the average values of A490 nm ≥0.2).

### Purification of MPER- and 6HB-specific antibodies from rabbit antisera

Two mg of MPER peptide (aa 659–683) or a recombinant 6HB mimetic, N36(L8)C34 were coupled to NHS-activated Sepharose 4 Fast Flow beads (GE Healthcare, Sweden) for purification of MPER- or 6HB-specific antibodies according to the manufacturer's instruction. Briefly, sera were diluted four times with PBS and passed through the affinity column. The column was washed with 20 ml PBS to remove nonspecific antibodies. The MPER- or 6HB-specific antibodies were eluted with low pH elution buffer (pH 2.5, 0.1 M Glycine). The purified antibodies were then concentrated and transferred to PBS by using a 30 kD ultra centrifugal filter device (Millipore, MA). The antibody concentrations were quantified by measuring absorbance at 280 nm and stored at 4°C until use.

### Assay for HIV-1 Env-mediated syncytium-formation

The assay for detecting the HIV-1 Env-mediated syncytium-formation was performed as previously described [24]. Briefly, (3T3.T4.CXCR4) cells (5×10^4^) were plated in 48-well plates, followed by an overnight incubation at 37°C and washed with GMEM-S medium. Then, 3×10^4^ effector (CHO-WT) cells pre-stimulated with 6.5 mM sodium butyrate for about 20 h were added in the presence of an antibody at a serial 2-fold dilution. After a co-culture at 37°C for 24 h, the syncytia, defined as giant cells with diameters more than four times larger than those of single cells, were counted under a microscope. The percentage inhibition of syncytium-formation was calculated as previously described [24], and the concentration for 50% inhibition (IC_50_) was calculated using the CalcuSyn software [25].

### HIV-1 neutralization assays

The neutralizing activity of antibodies was first determined using a single-cycle pseudovirus infection assay as previously described [26]. In brief, pseudoviruses were generated by transient transfection of HEK 293T cells with pNL4-3.LucR^−^E^−^ and an Env-expressing plasmid pHXB2 or pJRFL. The mixture of a pseudovirus at a concentration yielding 50,000–100,000 relative luminescence units (RLU) and a serial 2-fold dilution of an antibody (final concentration 4.7–150 µg/ml) was added to DMEM medium in wells of 96-well plates to reach a total volume of 100 µl. After incubation at 37°C for 1 h, 100 µl of Ghost cells (1×10^5^/ml) were added and cultured at 37°C in 5% CO_2_ for 48 h. RLUs were determined with a luciferase kit (Promega, Madison, WI) and luminometer (Ultra 386, Tecan, Durham, NC) following the instructions of the manufacturers.

The neutralizing activity of antibodies on infection by HIV-1 strain IIIB was determined as previously described [27]. Briefly, MT-2 cells (1×10^4^/well) were infected with HIV-1 IIIB at 100 TCID_50_ (50% tissue culture infective dose) in the absence or presence of 2-fold serially diluted antibodies in 200 µl RPMI medium 1640 containing 10% FBS overnight. The supernatants were removed, and fresh medium was added the next day. On the fourth day post-infection, 100 µl of culture supernatants from each well were collected and mixed with equal volumes of 5% Triton X-100 for testing p24 antigen using an in-house ELISA previously described [27].

The neutralizing activity of antibodies on infection by HIV-1 R5 strain Bal and primary isolates was performed as described before [28]. Briefly, 100 µl of TZM-bl cells (1×10^5^/ml) were pre-cultured overnight, followed by addition of a mixture of a virus at 100 TCID_50_ in the absence or presence of a serially diluted antibody. After co-culture at 37°C for 48 h, the cells were harvested and lysed by the lysing reagent in the luciferase assay kit. The RLUs were determined with luciferase kit and luminometer as described above. The percent of inhibition and the IC_50_ values were calculated with the CalcuSyn software as described above.

### Assessment of inhibition of antibodies on HIV-1 transmission from PBMCs to CEM×174 5.25M7 cells

Inhibitory activity of antibodies on HIV-1 transmission was assessed as previously described [29]. In brief, PHA/IL-2-stimulated PBMCs were infected by HIV-1 IIIB and Bal (a multiplicity of infection of 0.01) for 7 days and washed with culture medium for 3 times to remove free viral particles. Subsequently, 50 µl of HIV-1- infected PBMCs (1×10^6^/ml) were incubated with 50 µl of antibodies at 37°C for 30 min. Then, 100 µl of CEMx174 5.25M7 cells (5×10^6^/ml) were added and co-cultured at 37°C for 72 h. The cells were collected and lysed for analysis of luciferase activity, using a luciferase assay kit (Promega) as described above.

## Results

### Expression, purification, and characterization of NCM and its mutants

**Figure 2:**
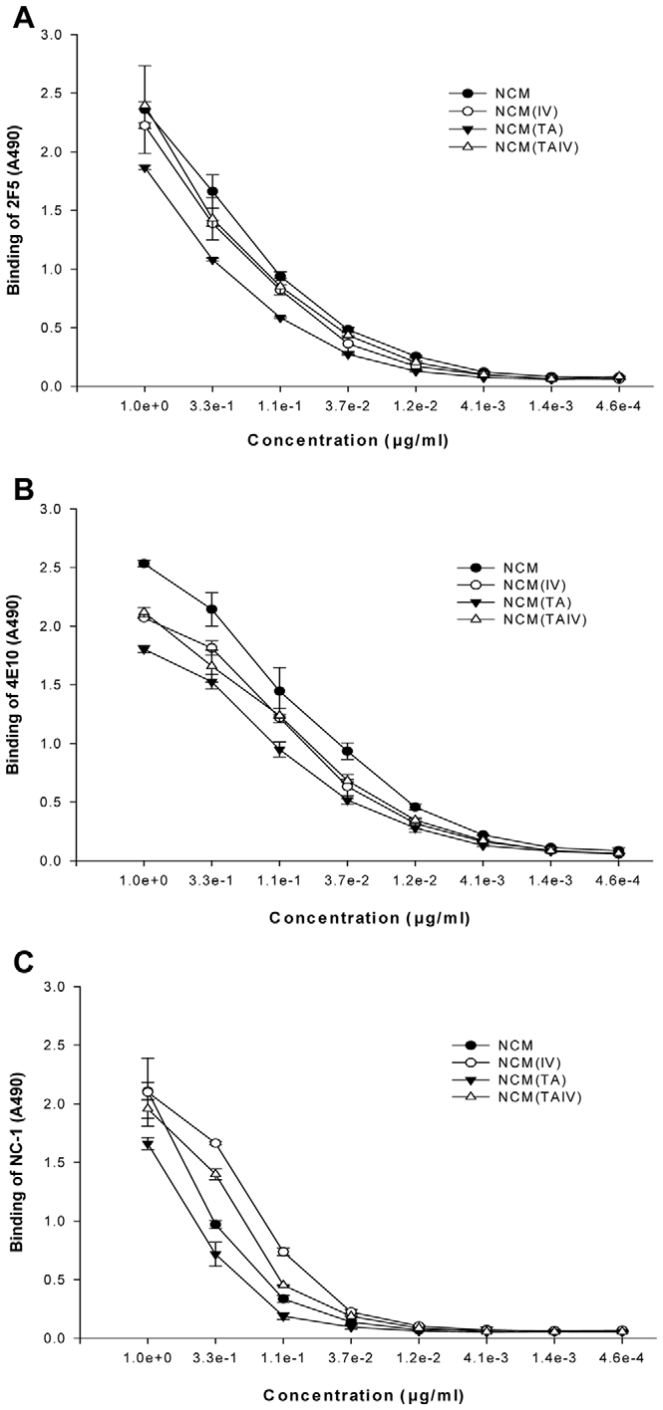
Binding of anti-gp41 mAbs to NCMs. Binding of human mAb 2F5 (A), 4E10 (B) and mouse mAb NC-1 (C) to NCMs, as determined by ELISA.

NCMs, including non-mutated NCM and three mutants, NCM(TA), NCM(IV) and NCM(TAIV) (Fig. 1A), were composed of NHR, CHR and MPER region derived from gp41 of a clade B strain HXB2. NCMs were expressed in *E. coli* and purified by HPLC with C4 preparative column. Purified NCMs appeared as a single band on SDS-PAGE at ∼12 kD (Fig. 1D). Chemical cross-linking of NCM with increasing concentration of glutaraldehyde (GA) resulted in the appearance of two new bands at ∼24 kD (dimer) and ∼36 kD (trimer) (Fig. 1E). NCMs were further characterized by ELISA using two MPER-specific neutralizing mAbs (2F5 and 4E10) [5], [7], [9], [13], [14] and a 6HB-specific mAb (NC-1) [22]. Similar to the NCM with wild-type sequence, we found that all the mutated NCMs could react with both MPER- and 6HB-specific mAbs in a dose-dependent manner (Fig. 2). These results suggest that each of these NCMs presents in solution mostly as a trimer of heterodimer (i.e., 6HBs) with three exposed MPER tails (Fig. 1C).

### NCM variant with double mutations, NCM(TAIV), elicited higher titers of MPER-specific antibody responses in rabbits than NCM and its variants with single mutation, NCM(TA) and NCM(IV)

**Figure 3:**
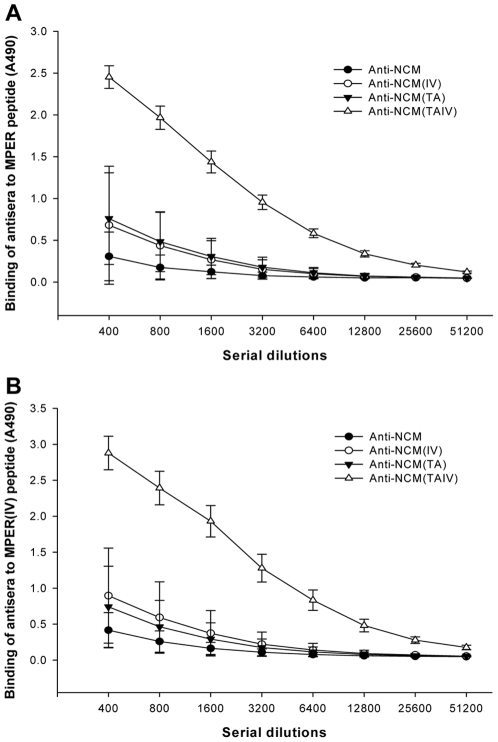
Binding of antisera to MPER or MPER(IV) peptide. Two-fold serially diluted rabbit sera were added to peptide MPER- (A) or MPER(IV) (B) -coated 96-well plates. Each datum is the average of A490 value of three rabbits in the same group.

To compare the immunogenicity of the four NCMs, which show one or two amino acid difference, the rabbit antisera induced by NCMs were evaluated by testing their binding to two MPER-peptides (Table 1). Although all rabbits generated high titers of antibodies against NCMs, the immunogenicity of MPER in different NCMs varied tremendously. The antiserum induced by NCM(TAIV) showed much higher titers of MPER-specific antibodies (1∶12800–1∶25600) than non-mutated and two other single- mutated forms (Table 1 and Fig. 3), which indicated that, among the four NCMs, NCM(TAIV) produced the best induction of MPER-specific antibodies.

### MPER-specific antibodies purified from anti-NCM(TAIV) sera exhibited broad neutralizing activity

**Figure 4:**
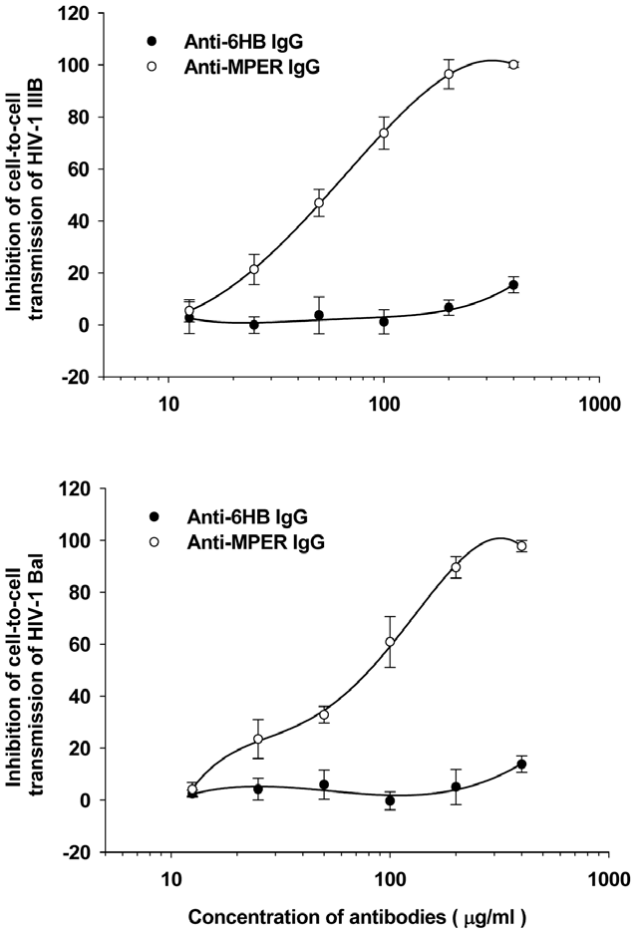
Inhibitory activity of antibodies on cell-to-cell transmission of HIV-1. Anti-MPER IgGs and ani-6HB IgGs were tested for inhibition of HIV-1 IIIB and Bal transmission from PBMCs to CEMx174 5.25M7 cells.

To evaluate the anti-viral activity, all antisera were tested in a pseudovirus assay using Ghost cells which express CD4, CCR5 and CXCR4. First, a sensitive pseudovirus assay to detect neutralizing antibodies (NAbs) was performed [26], [30]. The antisera to NCM(TAIV) could neutralize 50% of pseudovirus expressing HXB2 or JRFL envelope proteins at a dilution of 1∶20. On the other hand, no antisera exhibited 50% neutralization in a TZM-bl assay using HIV-1 isolate Bal or a p24 assay using isolate IIIB at the same dilution. The purified MPER-specific antibodies (anti-MPER) showed significant neutralizing activity to HXB2 and JRFL in a pseudovirus assay, as well as in an Env-mediated syncytium-formation, with IC_50_ values all below 25 µg/ml (Table 2). When tested in assays using PBMC-derived viruses, MPER-specific antibodies could neutralize isolates IIIB and BaL and several primary isolates, including 92US657 (subtype B), 93MW959 (subtype C) and 92TH009 (subtype A/E), with IC_50_ values below 150 µg/ml, but failed to neutralize primary isolates 94UG103 (subtype A) and RU570 (subtype G) (Table 2). In contrast, no significant neutralizing activity was shown by the 6HB-specific antibodies (anti-6HB) in any of the assays. Since cell-to-cell spread of HIV-1 plays an important role in HIV-1 dissemination [31], an assay was performed to determine whether MPER-specific antibodies could inhibit cell-to-cell transmission of HIV-1. In this assay, PBMCs infected by HIV-1 IIIB or Bal were co-cultured with CEMx174 5.25M7 cells in the presence or absence of MPER-specific antibodies. As shown in Fig. 4, MPER-specific antibodies, but not 6HB-sepcific antibodies, blocked cell-to-cell transmission of HIV-1 IIIB and Bal strains with IC_50_ of 56.4±7.2 and 79.2±8.1 µg/ml, respectively, suggesting that the MPER-specific antibodies are effective in blocking cell-to-cell transmission of HIV-1.

**Table 2:** Neutralizing activity of anti-MPER antibodies against Env-mediated syncytium-formation and infection by HIV-1 pseudoviruses, laboratory-adapted and primary strains.

HIV-1 isolates	Subtypes	Coreceptor tropism	IC_50_ (µg/ml)				
			Anti-6HB IgG	Anti-MPER IgG		2F5	4E10
Env-mediated syncytium-formation			>150	8.7±3.8		6.7±2.9	21.0±12.4
HIV-1 pseudoviruses							
HXB2	B	X4	>150	15.2±3.3	1.0±0.1		0.6±0.3
JRFL	B	R5	>150	23.8±6.4	1.4±0.2		3.9±2.1
Laboratory-adapted HIV-1 strains							
IIIB	B	X4	>150	64.6±16.8	13.2±3.7		8.3±1.6
Bal	B	R5	>150	79.6±2.6	16.1±5.1		9.2±1.1
Primary HIV-1 isolates							
94UG103	A	X4R5	>150	>150	27.1±4.3		41.8±8.3
92US657	B	R5	>150	142.1±27.7	10.2±2.9		7.5±3.5
93MW959	C	R5	>150	140.5±18.9	37.5±5.6		7.1±1.7
RU570	G	R5	>150	>150	17.2±8.2		25.1±7.6
92TH009	A/E	R5	>150	98.2±11.5	6.3±1.1		3.5±0.9

### Potential mechanism to explain improved immunogenicity of NCM(TAIV)

**Figure 5:**
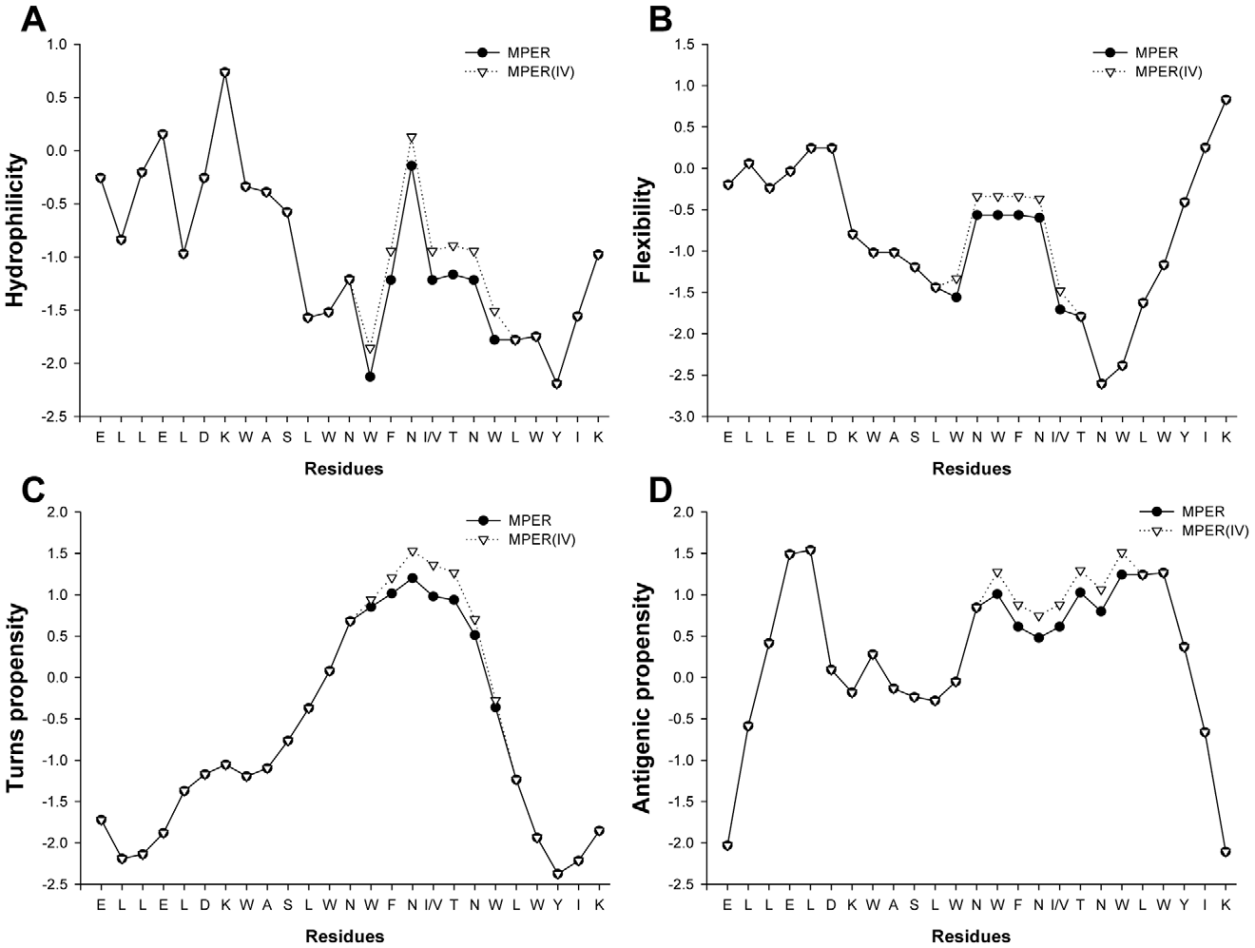
Immunoinformatic analysis of physicochemical properties of MPER. (A) Hydrophilicity, (B) Flexibility, (C) Turn propensity and (D) Antigenic propensity. All analyses were performed by BcePred server.

To understand why NCM(TAIV) exhibited higher immunogenicity than NCM, NCM(TA) and NCM(IV), we used an immunoinformatic method to predict the continuous B-cell epitopes on MPER based on recurrent neural network [32]. The results indicated that two epitopes existed in the MPER of gp41, including ELDKWASLWN and SLWNWFNITN, with scores of 0.69 and 0.50, respectively. These two epitopes actually overlap the epitopes for the human broad neutralizing mAbs 2F5 (ELDKWA) and 4E10 (NWFNIT), respectively [5], [7], [9], [13], [14]. Interestingly, the mutated epitope SLWNWFNVTN (I675V) exhibited a higher score (0.54) than the non-mutated epitope, suggesting that this sequence has a higher probability of functioning as an antibody epitope by this mutation. Subsequently, we analyzed the physicochemical property changes caused by mutation I675V with BcePred server [33]. As expected, the I657V mutation could increase the antigenic propensity of the NWFNITNW region because of the elevated hydrophilicity, flexibility and turns (Fig. 5). We could not predict the potential influence of T569A mutation on the immunogenicity of NCM(TAIV) using these methods because this residue is far away from the epitopes in the MPER of gp41.

**Figure 6:**
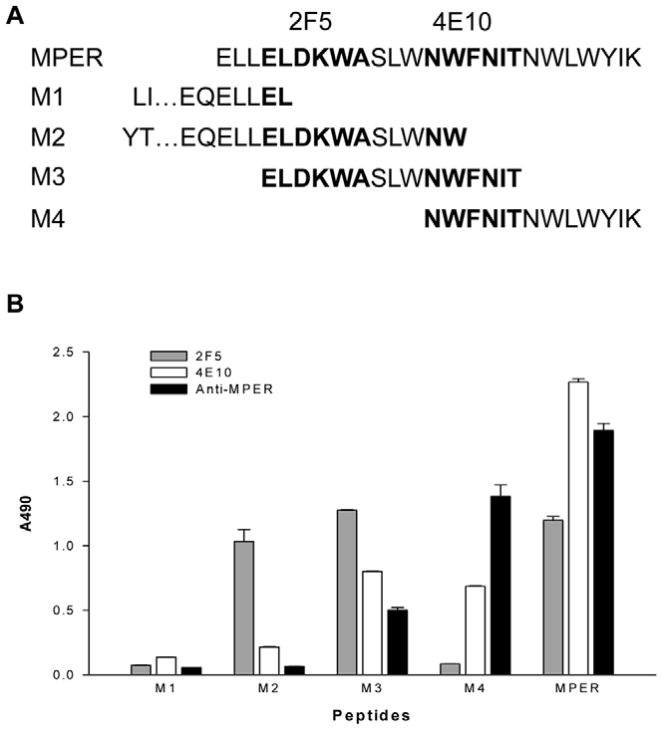
Exploring binding sites of anti-MPER. (A) Schematic representation of peptides used for scanning the binding sites of anti-MPER. (B) Results of anti-MPER binding to peptides in ELISA. MAbs 2F5 and 4E10 were used as control. The concentration of each antibody was 0.2 µg/ml.

To further define the binding site(s) of the NCM(TAIV)-induced antibodies with neutralizing activity, we synthesized 5 peptides, including MPER and M3 (which contain both 2F5 and 4E10 epitopes), M2 and M4 (containing 2F5 and 4E10 epitopes, respectively), and M1 (containing neither 2F5 nor 4E10 epitope) (Fig. 6), and used these peptides to determine the binding specificity of the purified MPER-specific antibodies with ELISA. As shown in Fig. 6, mAb 2F5 bound to MPER, M2 and M3 peptides, while mAb 4E10 recognized MPER, M3 and M4 peptides. Similar to 4E10, the MPER-specific antibodies induced by NCM(TAIV) strongly reacted with MPER, M3 and M4 peptides that contain the NWFNIT-epitope, but showed only marginal binding activity to M2 peptide containing the ELDKWA-epitope. These data indicate that the immunogen NCM(TAIV) has increased immunogenicity to induce antibodies against the 4E10 epitope.

**Figure 7:**
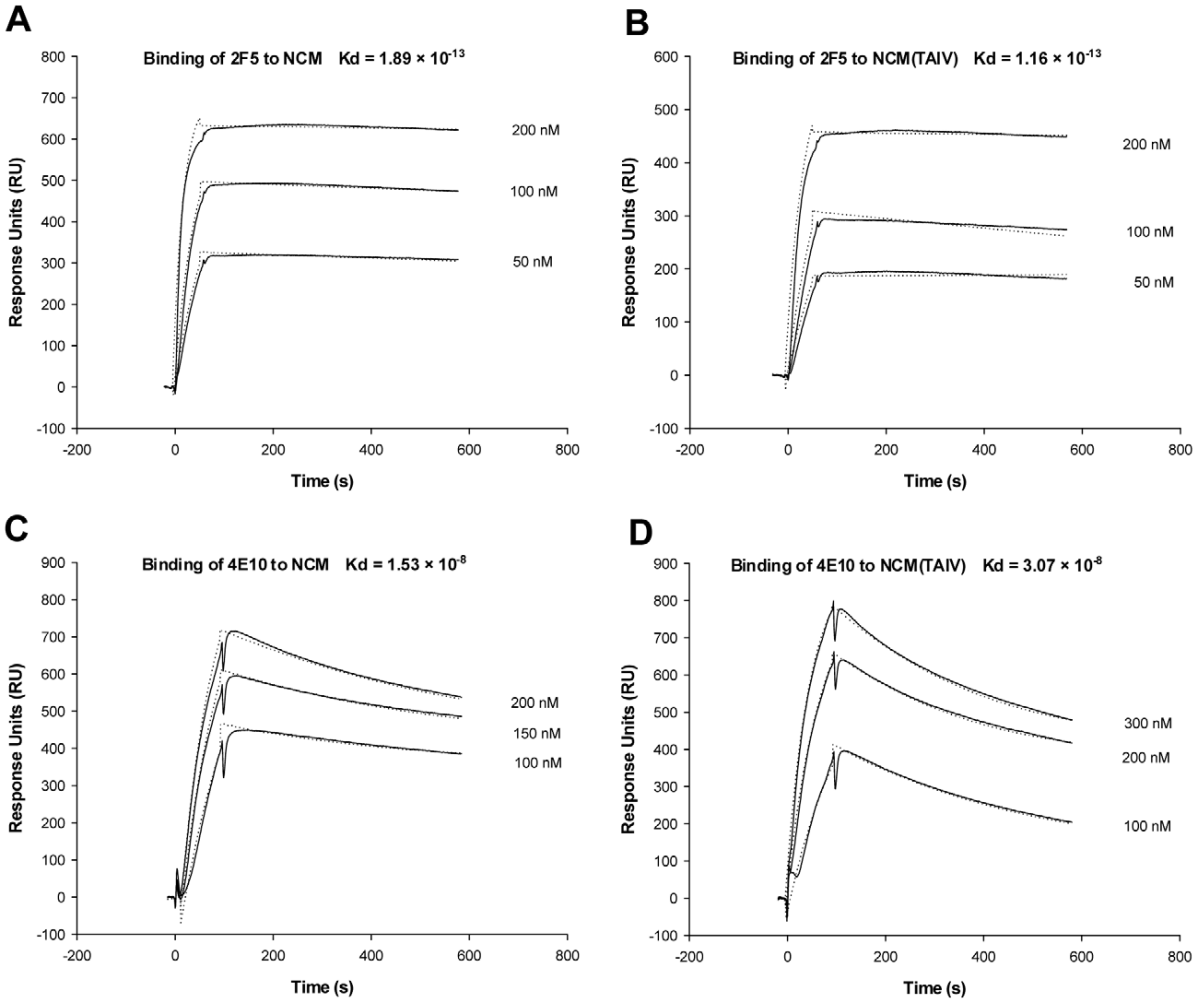
Kinetic analysis of mAbs binding to NCM captured on membranes. (A) Sensorgrams obtained for 2F5 binding to NCM or NCM(TAIV) captured on membranes. (B) Sensorgrams obtained for 4E10 binding to NCM or NCM(TAIV) captured on membranes. Experimental curves (solid line) are shown overlaid with fitted curves (dash line) obtained with the 1∶1 binding with drifting baseline model.

**Figure 8:**
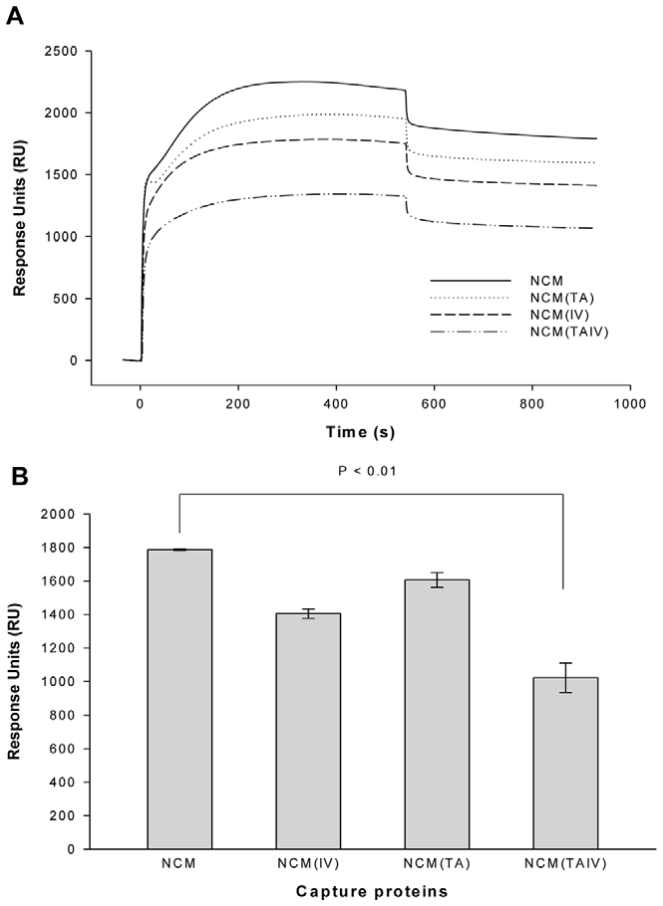
Capture levels of NCMs binding to membrane. (A) Sensorgrams obtained for 1 µM NCMs binding to POPC/Chol/SM membranes on the L1 sensor chips. (B) Different capture levels of NCMs (RUs at 400s post-injection).

Surface plasmon resonance (SPR) assay was utilized to evaluate the kinetics and affinity of mAbs 2F5 and 4E10 for binding to NCMs immobilized on membranes since membrane was suggested to be a crucial component of 2F5 and 4E10 epitopes. As shown in Fig. 7, there was no significant difference in kinetics and affinities of 2F5 and 4E10 for binding to NCM and NCM(TAIV). Interestingly, however, the capacity of NCM(TAIV) captured into the membranes was significantly lower than that of NCM and its analogs with single mutation (Fig. 8), thus possibly explaining why NCM(TAIV) has higher immunogenicity than NCM and other NCM mutants.

## Discussion

We and others have made extensive efforts and used different strategies to induce neutralizing antibodies in vaccinated animals using MPER-containing peptides, recombinant proteins or surface antigen of human hepatitis B virus (HBV). Thus far, however, no one has been able to elicit potent and broad neutralizing antibodies, like 2F5 or 4E10, targeting the gp41 MPER region [34], [35], [36], [37], [38], [39], [40], [41], [42]. This can be explained by the failure of neutralizing epitopes in the MPER-based immunogens to maintain the correct conformation. It is also possible that the gp41 NHR and CHR structures at the upstream of MEPR affect the conformation and immunogenicity of neutralizing epitopes in MPER. It follows that the immunogen should contain both MPER and other regions in the gp41 ectodomain with appropriate conformation.

In the present study, we used the gp41 ectodomain as the basic structure to design the MPER-based immunogens. First, we removed the fusion peptide (FP) from the immunogen since our previous studies have shown that FP suppressed the immunogenicity of MPER and that deleting FP resulted in enhanced antigenicity and immunogenicity of the 4E10 epitope [42]. Second, we replaced the immunodominant loop region with an 8-mer (GGGKLGGG) linker, since the loop region mainly elicits cluster I antibodies [43], which do not neutralize, but rather enhance, HIV-1 infection [44]. The resulting immunogen, contains the gp41 NHR, CHR and MPER domains, thus designated NCM. Based on the crystal structure of HR1-54Q, a similarly designed recombinant gp41 containing MPER [45], [46], NCM may also form 6HB core with an exposed MPER tail since it reacted strongly with the 6HB-specific mAb NC-1 [22] and the MPER-specific antibodies 2F5 and 4E10 [5], [7], [9], [13], [14] (Fig. 2). Finally, we introduced the T579A and/or I675V mutations into NCM to generate three NCM mutants, NCM(TA), NCM(IV) and NCM(TAIV) (Fig. 1A). It was reported that introduction of T659A/I675V mutations into gp41 of a neutralization-resistant HIV-1 variant significantly increased exposure of the conserved neutralization epitopes in MPER and resulted in 3- to 360-fold enhanced neutralization by 2F5 and 4E10 [19]. Therefore, we assumed that introduction of the T659A and/or I675V mutations into NCM might increase its immunogenicity for eliciting neutralizing antibodies.

Indeed, as expected, all antisera from the rabbits immunized with NCM(TAIV) had much higher titers of antibodies against the gp41 MPER domain (1∶16,127) than those from rabbits vaccinated with NCM (1∶504), NCM(TA) (1∶1008) or NCM(IV) (1∶1008) (Table 1 and Fig. 3), suggesting that introducing both mutations (T659A/I675V) could significantly enhance the immunogenicity of the NCM-based immunogen.

There are two major antibody populations in the rabbit antisera induced by NCM(TAIV) – the 6HB-specific and MPER-specific antibodies. We purified these antibodies using affinity columns conjugated with a recombinant 6HB protein and an MPER peptide, respectively, and compared their neutralizing activities. The 6HB-specific antibodies had no neutralizing activity at the concentration of >150 µg/ml, while the MPER-specific antibodies exhibited inhibitory activity against HIV-1 Env-mediated syncytium-formation and infection by HIV-1 pseudoviruses, laboratory-adapted and some primary HIV-1 isolates (Table 2). These results indicate that the altered epitopes in the gp41 MPER, rather than those in the gp41 6HB core, elicit neutralizing antibodies in vaccinated animals.

The precise mechanism by which these mutations exposed neutralization epitopes remained unclear, since chemical cross-linking assay, together with antibodies binding assays by ELISA and SPR, indicated no significant difference between NCM and mutants. However, several studies have demonstrated that strictly conserved T569 and I675 residues play important roles in viral entry. T569 residue is located in the inner face of NHR trimer and falls within the hydrophobic pocket which is critical for interaction with CHR. Therefore, T569A mutation may partially disrupt the gp41 6HB core conformation [19], resulting in change of the overall conformation of gp41. It was reported the I675V mutation could affect some physicochemical properties of MPER which contribute to increased immunogenicity. However, these changes could not fully explain the strong immunogenicity of MPER in NCM(TAIV), since single-mutated NCM(IV) did not elicit high titers of MPER-specific antibodies. Therefore, we considered the effect of another interesting finding that NCM(TAIV) showed lower capture level to membranes. Many studies reported that the membrane plays a crucial role in defining the structure of the MPER and, consequently, the formation of the 2F5 and 4E10 epitopes. The long CDR-H3s of these antibodies were involved in the interaction with lipids [47], [48]. However, the immersion of MPER in membrane may have a negative effect on immunogenicity of MPER because of the steric restrictions imposed by the viral membrane which may hamper the generation of antibodies. Although the rabbits were immunized with NCM and its mutants in the absence of liposomes, the immunogen could diffuse and nonspecifically interact with membrane systems in multiple immune processes after subcutaneous injections. This interaction may result in the decreased exposure of the epitopes on MPER, rendering a correspondingly poorer immunogenicity. On the contrary, decreasing the immersion depth of epitopes in membrane could contribute to an increase of immunogenicity. In fact, the 4E10 epitope was immersed in the polar/apolar interfacial region of the lipid bilayer, whereas 2F5 epitope was more solvent exposable [49]. Thus the exposure of 4E10 epitope might be more sensitive to immersion depth. I675 residue was reported to be one of the rare residues which immersed deeply both before and after 4E10 binding [10]. Therefore, a shorter side chain of Valine in I675V mutant may facilitate the decreasing immersion depth of MPER, especially the 4E10 epitope. Still, how T569A and I675V mutations synergistically affect the capture level of NCM(TAIV) remains to be further studied. We suggested that the moderate immersion depth of MPER in membranes, which made MPER more exposed, but antigenically preserved, was another reason underlying the ability of NCM(TAIV) to elicit higher titers of MPER-specific antibodies. Interestingly, the induced MPER-specific antibodies mainly targeted an extended 4E10 epitope as we had expected. Thus, physicochemical property and structure changes, together with decreased immersion depth, may correlate with the increased immunogenicity of MPER in NCM(TAIV).

Although NCM(TAIV) could elicit relatively higher titers of MPER-specific antibodies than non-mutated NCM or other gp41-derived recombinant proteins described previously, it lacks the ability to induce highly potent and broad neutralizing antibodies against primary HIV-1 isolates. Therefore, more efforts should be taken to make NCM(TAIV) a rational candidate for an HIV vaccine to elicit higher titer and more potent and broader neutralizing antibodies. Fortunately, many studies have provided some useful suggestions, such as implementing a new immunization strategy [15], using strong adjuvants to break B-cell tolerance [50], or introducing mutations which could favor the formation of prehairpin intermediate conformation or prolong exposure of MPER [51], [52].

In conclusion, our study presented a rationally designed immunogen consisting of the gp41 6HB core and the exposed MPER tail with a double mutation (T569A and I675V). This immunogen could elicit high titers of MPER-specific antibodies with broad neutralizing activity. Although the precise underlying molecular mechanism remained unclear, we confirmed that that double T569A/I675V mutations in gp41 are critical for significantly enhancing the immunogenicity of neutralizing epitopes in the gp41 MPER. Therefore, this study might provide important implications for designing novel MPER-based HIV-1 vaccines with increased immunogenicity for eliciting potent and broad neutralizing antibodies.
